# A deep dive into seagrass Rubisco catalytic properties uncovers a distinct evolutionary trajectory

**DOI:** 10.1093/plphys/kiac546

**Published:** 2022-12-06

**Authors:** Gustaf E Degen

**Affiliations:** School of Biosciences, University of Sheffield, S10 2TN, UK

Ribulose 1,5-bisphosphate carboxylase/oxygenase (Rubisco) is the most abundant and arguably most important enzyme on Earth, as it is the entry point of carbon into the biosphere. CO_2_ fixation by Rubisco onto ribulose-1,5 bisphosphate (RuBP) is the first step in the Calvin–Benson–Bassham cycle, resulting in two molecules of 3-phosphoglycerate (3PGA), which ultimately make up the biomass of photosynthetic organisms. Efficient CO_2_ fixation is hindered by the inability of Rubisco to discriminate between CO_2_ and O_2_. Oxygenation of RuBP leads to the production of toxic 2-phosphoglycolate, which is recycled back to 3PGA via photorespiration, consuming energy and releasing CO_2_ in the process (Sage et al., 2012). In agriculturally important plants such as wheat (*Triticum aestivum*) or rice (*Oryza sativa*), photorespiration can cause losses greater than 25% of the CO_2_ assimilated by photosynthesis (Walker et al., 2016), making Rubisco a key target for improving crop photosynthesis.

Rubisco activity and efficiency are measured by determining multiple parameters. The carboxylation velocity (*V*_c_) refers to the CO_2_-fixation activity per leaf area and is arguably the most important parameter. The turnover number (*k*_cat_) is the amount of product per active site per second. The Michaelis–Menten constants (*K*_m_) for CO_2_ and O_2_ (*K*_c_, *K*_o_) refer to the affinity of Rubisco to CO_2_ and O_2_, respectively. A higher *K*_m_ means that more substrate is required to reach half of the maximum enzyme velocity, suggesting a lower affinity of the enzyme for the substrate. Finally, the specificity factor for CO_2_ and O_2_ (*S*_C/O_) is a measurement of the ability of Rubisco to discriminate between CO_2_ and O_2_, where a higher value indicates that Rubisco binds more CO_2_ relative to O_2_.

Surveying Rubisco kinetic diversity has received much attention in recent years. Analysis of Rubisco catalytic properties between the C3 plant wheat and its wild relatives uncovered a large diversity in Rubisco efficiencies (Prins et al., 2016). Comparing C3 plants to C4 plants, which evolved a carbon-concentrating mechanism (CCM) around 25 million years ago (mya; Sage et al., 2012), showed that *k*^c^_cat_ in C4 plants was superior, whereas SC/O was lower (Sage, 2002; Orr et al., 2016; Iñiguez et al., 2020). Due to the enrichment of CO_2_ in C4 plants, the CO_2_ specificity can be lower, allowing for higher rates of catalysis. This shows the impact CCMs have on the catalytic adaption of Rubisco.

**Figure 1:**
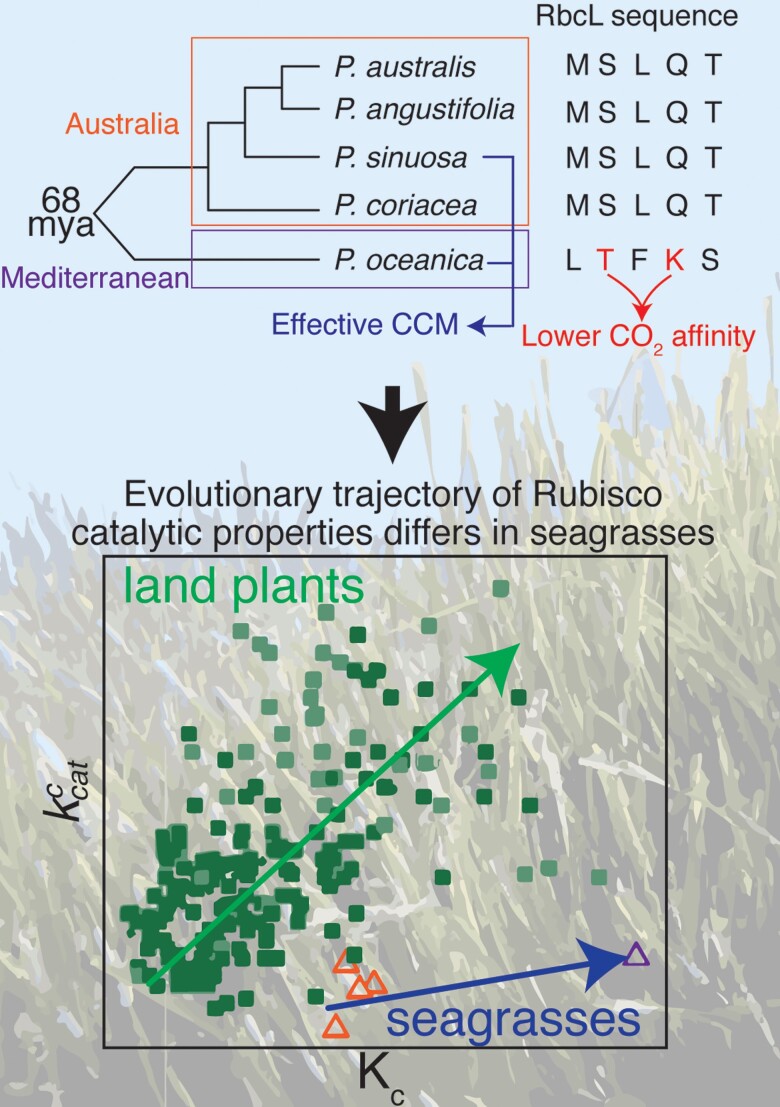
Australian and Mediterranean *posidonia* (seagrass) Ribulose 1,5-bisphosphate carboxylase/oxygenase (Rubisco) catalytic properties. Australian and Mediterranean species diverged 68 mya. Analysis of the large Rubisco subunit (RbcL) and Rubisco properties revealed that the *Posidonia oceanica* species had two amino acid changes that contributed to a higher Michaelis–Menten constant for CO_2_ (*K*_c_). Furthermore, only two species were found to have an effective CCM due to adaption to their environment. Analyzing the relationship between *K*_c_ and turnover number for CO_2_ (*k*^c^_cat_) showed a distinct evolutionary trajectory of seagrasses, in contrast to land plants. Background image adapted from ‘Posidonia oceanica observed at La Ciotat’ by Frédéric Ducarme is licensed under CC BY-SA 4.0. Other figures adapted from Capó-Bauçà et al. (2022).

To further the understanding of Rubisco evolution, marine environments are useful because of the slow diffusion of CO_2_. The limited supply of CO_2_ to Rubisco in marine plants, such as seagrasses (*Posidonia*), has also resulted in the invention of CCMs, such as bicarbonate transporters. However, little is known about the evolutionary trajectory of catalytic properties in seagrasses, which colonized the oceans some 100 mya.

In this issue of *Plant Physiology*, Capó-Bauçà et al. (2022) investigated the kinetic properties of Rubisco in seagrass species from the Mediterranean and seas around Australia, revealing that these follow a different evolutionary trajectory compared to Rubiscos found in terrestrial angiosperms.

In the first set of experiments, the authors characterized Rubisco affinity for CO_2_ and showed that *K*_c_ was much higher for the Mediterranean seagrass species, meaning that it had a lower affinity for CO_2_ than the Australian species. Interestingly, the *K*_o_ of all seagrass species was much higher than those reported for other angiosperms, diverging from the linear relationship between *K*_c_ and *K*_o_ observed in the terrestrial plant Rubiscos. Surprisingly, the slower turnover rate in seagrasses was also accompanied by lower CO_2_ affinity, in contrast to land plant Rubiscos. However, the O_2_ affinities in seagrasses were also lower, which balances out the high *K*_c_. Similar to other Rubiscos found in cyanobacteria or red algae (Shih et al., 2016), the authors concluded that seagrass Rubisco catalytic properties did not follow the same coevolutionary trend as terrestrial angiosperms.

This insight prompted the authors to investigate the amino acid sequence of the large Rubisco subunit (RbcL), which hosts the active site of Rubisco. They identified five amino acid changes in the Mediterranean species, which diverged 68 mya from the Australian relatives. Considering the location of these residues in the holoenzyme, the authors identified positions 279 and 449 as potentially accounting for the lower CO_2_ affinity in the Mediterranean species. This also revealed that all Australian species shared the same RbcL sequence, suggesting that the observed low *k*^c^_cat_ of one of these species is likely due to sequence differences in the small Rubisco subunit.

Not only did the seagrass species differ in their Rubisco catalytic properties but they also varied in the effectiveness of their CCMs. This was shown when using Tris-buffered seawater where photosynthetic activity depended on the efficiency of the CCM. In fact, only two out of the five species studied had an effective CCM, suggesting adaptation to the natural environment where light levels may influence the rate of energy production used to fuel CCMs. Thus, in some cases, an effective CCM is required, whereas for plants growing in deeper water, lower light, and higher CO_2_ partial pressure, a less effective CCM is advantageous.

Overall, Capó-Bauçà et al. (2022) show that the evolution of seagrass Rubiscos in the ocean lead to different but advantageous changes in catalytic properties that are distinct from terrestrial angiosperm relatives (summarised in Figure 1). Their findings add to the growing body of work on Rubisco kinetic diversity, which is a valuable tool to improve Rubisco properties.
